# Supplementary material for: Asynchronous magnetic resonance elastography: Shear wave speed reconstruction using noise correlation of incoherent waves
Source: Magn Reson Med. 2022 Oct 27;89(3):990–1001. doi: 10.1002/mrm.29502 (PMC9792433; doi:10.1002/mrm.29502)
Supplement: Supplementary file 1 — DATA S1 MATLAB code used to generate the numerical simulations of Figure 1 [file MRM-89-990-s002.zip › k-Wave/helpfiles/voxelPlot.html]

voxelPlot :: Functions (k-Wave)


# voxelPlot

3D plot of voxels in a binary matrix.

## Syntax

```
voxelPlot(mat)
voxelPlot(mat, ...)
```

## Description

`voxelPlot` produces a 3D plot of a binary matrix, where filled voxels are displayed at the positions of the 1's. The colormap, transparency, and axis limits can be controlled through optional inputs. For example running

```
voxelPlot(makeBall(20, 20, 20, 10, 10, 10, 4), 'AxisTight', true, 'Color', [1 0 0], 'Transparency', 0.5);
view([50, 15]);
```

will produce a semi-transparent image of a red ball.

## Inputs

|  |  |
| --- | --- |
| `mat` | binary 3D matrix |

## Optional Inputs

Optional 'string', value pairs that may be used to modify the default computational settings.

| Input | Valid Settings | Default | Description |
| --- | --- | --- | --- |
| `'AxisTight'` | *(Boolean scalar)* | `false` | Boolean controlling whether axis limits are set to only display the filled voxels. |
| `'Color'` | *(three element numeric vector)* | `[1, 1, 0.4] (yellow)` | Three element vector specifying rgb color. |
| `'Transparency'` | *(numeric scalar)* | `0.8` | value between 0 and 1 specifying transparency (1 gives no transparency). |

## Examples

- Simulations In Three Dimensions
- Focussed Detector in 3D
- Modelling Sensor Directivity in 3D
- 3D FFT Reconstruction For A Planar Sensor
- 3D Time Reversal For A Spherical Sensor

## See Also

`patch`
